# Behavior in Avalanche Terrain: An Exploratory Study of Illegal Snowmobiling in Norway

**DOI:** 10.3390/ijerph19106040

**Published:** 2022-05-16

**Authors:** Bjørn Michaelsen, Iain Stewart-Patterson, Carsten G. Rolland, Audun Hetland, Rune V. Engeset

**Affiliations:** 1 School of Sport Sciences, Faculty of Health Sciences, UiT the Arctic University of Norway, 9037 Tromsø, Norway; carsten.g.rolland@uit.no (C.G.R.); rue@nve.no (R.V.E.); 2 Adventure Studies Department, Thompson Rivers University, Kamloops, BC V2C 0C8, Canada; iainsp2010@gmail.com; 3 Department of Psychology, Faculty of Health Sciences, UiT the Arctic University of Norway, 9037 Tromsø, Norway; audun.hetland@uit.no; 4 Norwegian Water Resources and Energy Directorate, 0103 Oslo, Norway

**Keywords:** avalanche education, qualitative method, illegal, snowmobiling, persistent weak layers

## Abstract

Snowmobilers make a grim and significant contribution to avalanche fatality statistics in Norway. However, there is limited knowledge on the behavior of this group in avalanche terrain and the factors influencing this behavior. Our study documents what snowmobilers do and not do in avalanche terrain, how their behavior relates to managing complex avalanche conditions and if there is a mismatch between avalanche competence, education and riding preferences. This ethnographic study observed snowmobiler tracks and thus avalanche terrain usage in Northern Norway during 2018 and 2019, supported by open-ended conversations with target group riders. Results show that high-marking lost popularity to technical riding, which seems to be perceived as safer despite increased exposure to complex avalanche terrain and conditions with persistent weak layers in the snowpack. The detected mismatch between preferences and avalanche knowledge/attitude will remain an obstacle to future accident prevention efforts unless behavioral changes are addressed. This study of a predominantly illegal activity sheds light on how to explore and observe hard-to-reach illegal activities and should be of interest to a wider audience from other research disciplines.

## 1. Introduction

Snowmobilers’ behavior in avalanche terrain is causing them to get killed in avalanches [1,2,3]. In most recreational avalanche accidents, the avalanche is triggered by the victim or someone in their party [4]. In response, new research and methods from social science are requested to better understand the human factors that determine decision making in avalanche terrain [5,6,7,8,9]. Even though there is a growing body of literature on human factors in avalanche terrain, studies of what snowmobilers actually do in the field is sparse.

Despite the effort from Canadian and US avalanche education to use snowmobilers to educate snowmobilers the past 20 years [10,11,12,13], avalanche education has been questioned regarding its ability to adapt to changing circumstances within snowmobiling [14,15]. A rethinking of avalanche education has been suggested in Canada due to a likely mismatch between the snowmobilers’ behavior and education since more riders have easier and increased access to more complex management situations [15,16,17,18].

In Norway, snowmobilers accounted for 23% of the avalanche accidents from 2009–2019. We do not know if there is a mismatch between competence and snowmobilers’ preferences, as has been reported in Canada [17,18]. However, we know that complex snowpack situations with persistent weak layers dominate Norwegian avalanche accidents [19], as in Canada [20]. Risks that are difficult to manage even for skilled avalanche professionals [21].

Riders in avalanche terrain are difficult to enlist in regular studies given the fact that this activity is mostly illegal in Norway. Survey-based studies from Canada and the USA [11,15,22] have contributed substantial knowledge about snowmobilers, but have also called for fieldwork that can deepen our understanding of actual behavior [22]. This is the aim of this research.

To study these hard-to-reach snowmobilers we used an exploratory qualitative approach, where observations of behavior were validated through ethnographic interviews with key participants. This research may be used to improve mitigation strategies and benefit educators, practitioners, policymakers and researchers in the quest of avalanche accident prevention among snowmobilers.

## 2. Background

The avalanche community called for more collaborative efforts with behavioral science researchers during the 2016 International Snow Science Workshop (ISSW) at Breckenridge [18]. Behavioral theory tells us that just sharing rational expert knowledge to the less knowledgeable is looked upon as insufficient if your aim is to change behavior [23]. According to Darnton [24] (pp. 31–33) you have to “understand the target behavior and the factors influencing it from the audiences’ perspective” if you want to accomplish behavioral change.

Snowmobile avalanche education in Canada and the US has decades of experience trying to influence snowmobilers’ behavior in avalanche terrain [13], and there is increasing knowledge about factors that are important [9,22]. However, Canadian snowmobile avalanche education has been questioned by Stewart-Patterson and Hanke [16,20], who suggested a rethink of the avalanche education as it was not keeping up with the changing riding preferences in avalanche terrain. They argued that an increasing number of people with limited experience, skills and knowledge were obtaining increasingly easier access to complex management situations due to the emerging technology that made snowmobiling easier.

This may also apply to Norway. To place this study in context, we give a more detailed background on snowmobiling, regulations, avalanche terrain, riding styles, avalanche problems and avalanche education.

Snowmobiling is using a motorized vehicle for winter travel and recreation on snow and does not require a road or trail [25]. A snowmobiler can also be referred to as a rider or sledder. When you leave the trail in the backcountry, snowmobiling is called freeriding. Riding off the tracks demands increasing personal skills and machine performance due to varied snow characteristics, the risk of getting stuck, hitting obstacles and avalanche danger. Recreational snowmobiling in Norway has been restricted to official trails since 1978, permitting a 30 m deviation alongside the trail and up to 300 m from the trail for rest stops only [26]. The official trails normally avoid avalanche terrain, thus freeriding in avalanche terrain is mostly considered illegal.

Avalanche terrain is usually defined as terrain steeper than 30 degrees [3]. In addition to the direct triggering when riding in steep enough terrain, riders can also indirectly endanger themselves and others in the less steep runout areas due to weak layers in the snowpack that can propagate up to the stepper terrain and starting zones of the avalanches.

**Figure 1:**
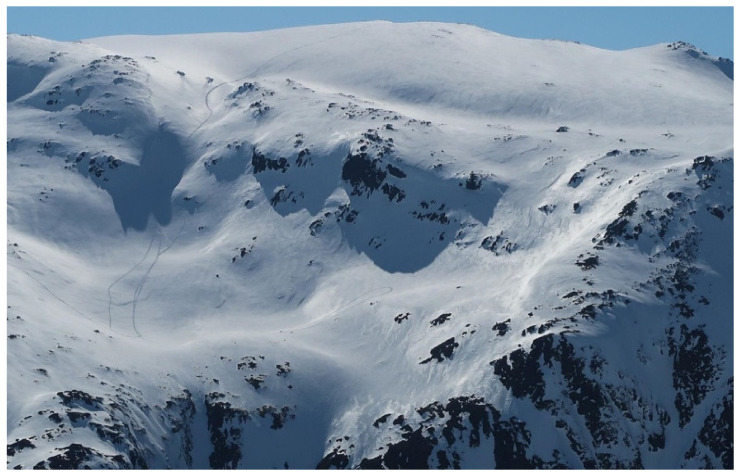
Typical terrain used for high-marking and hill climbing. Photo: Courtesy of participant.

**Figure 2:**
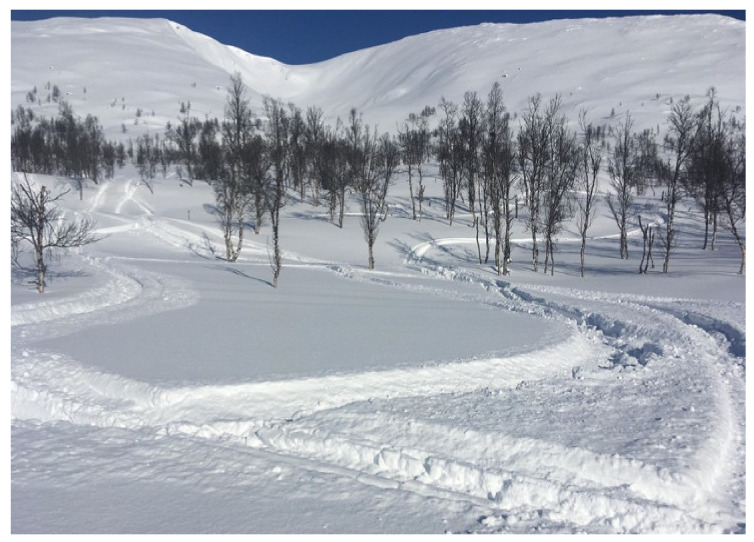
Technical riding tracks in the focus area between the trees. Note that there are no tracks in the classic hill climbing bowl in the background. Photo: B. Michaelsen.

Various freeriding styles use avalanche terrain differently [27]. Three freeriding styles have evolved during the technical evolution of snowmobiles. The first two, high-marking and hill climbing (Figure 1), typically start at the bottom of a slope and climb as high as possible to create a competitive “high mark” or climb the whole mountain without turning. High-marking has been a precipitant for numerous avalanche accidents [28] and has dominated mitigation focus [10]. During the past 15 years, a third riding style has evolved, termed technical riding (also known as boon-docking). It prefers deep loose snow to make tight turns, jumps, drops, short climbs and slope traversing/cutting, as shown in Figure 2 [29,30]. During technical riding, fellow riders are often out of sight [31] of each other, resulting in poor internal communication.

The most complex avalanche management situation is when there are persistent weak layers (PWLs) in the snowpack. According to Klassen, “At no time do human factors play a greater role in decision making than when dealing with persistent slab, and especially deep persistent slab, avalanche problems” [21] (p. 175). PWLs are fragile snow layers within the snowpack that can exist over longer periods in large areas and imply a greater uncertainty than other avalanche problems, regarding if and when they might collapse and trigger an avalanche. They can be triggered naturally or by human activity in steep slopes or remotely from flat areas below steeper avalanche terrain. PWLs are evident in most avalanche accidents in Norway, e.g., the winter of 2018–2019 [32], and are a causal factor in 60–75% of avalanche fatalities in Canada [20]. The high rate of accidents due to PWLs highlights the need for improved strategies for managing this avalanche problem.

Snowmobiling can have a compacting and thus stabilizing effect on snow and therefor reduce the avalanche danger if an area experiences heavy traffic. Compaction may reduce or eliminate the PWL’s ability to propagate and create an avalanche [33]. Snowmobiles penetrate the snowpack deeper than skiers [34], especially when performing technical maneuvers while freeriding [35]. This stabilizing effect is exploited in ski resorts, where bootpacking is used to prevent inbound avalanches [33,36]. The benefit of compaction is probably greater when backcountry riding is frequent between snowfalls. Snowmobilers may get fewer alarm signs from PWLs in areas more prone to snowmobiling and compaction. Firm layers protecting the deeper PWL layer can provide a false sense of safety. All three riding styles imply extreme maneuvers with unpredictable effects on PWLs [35], and thus demand more experience and a higher level of avalanche knowledge compared to riding in thoroughly compacted areas [20].

Snowmobile avalanche education started about 30 years ago [10]. Mainstream avalanche books have primarily targeted skiers (see [37] for example). Snowmobiling and high-marking strategies became more noticeable in avalanche books in early 2000 in e.g., *Staying Alive in Avalanche Terrain* [37]. Norwegian snowmobilers were addressed (2 out 86 pages) by Brattlien [38]. The increasing amount of snowmobile avalanche accidents 20 years ago in the US and Canada put more focus on communication challenges regarding skier–snowmobile perspectives. A shift toward an audience perspective was applied by using snowmobilers to educate snowmobilers [10,12]. They tried to take advantage of social comparison theory [39] so that snowmobilers would increasingly identify themselves with the need to obtain avalanche education and not only the riding skills of professional riders. The aim was that snowmobilers would take courses and know how to adjust their behavior [15] according to the avalanche danger. In Norway, the Norwegian Avalanche Warning Service started a snowmobile educational web site in 2015 [40], and the Norwegian snowmobile license education introduced a six-hour avalanche and ice safety course in 2018 [41] where the final section of the program implied basic snow knowledge, identification of avalanche terrain, how avalanche danger is influenced by snowmobiling and search and rescue. Riding styles and challenges specific for freeriding are not mentioned, probably due to the illegal aspects of this activity.

Snowmobilers’ avalanche knowledge has been studied and was rated as low from a snowmobiler trail head survey in Canada [42]. Further research on snowmobilers’ knowledge regarding complex snowpack management was also rated low [10]. Haegeli and Strong-Cvetich’s [22] online survey in Canada found a misunderstood use of the avalanche danger scale, low perception of PWLs, the need to emphasize more on warning signs and the importance of relocation strategies at elevated danger. Despite the efforts made in Canada and the US [13], snowmobilers do not seem to take courses beyond the basics [16,17]. Since complex management situations with PWLs are not taught at introductory courses, [20] and [43] emphasized that snowmobilers could develop overconfidence in avalanche terrain. The authors of [16,20] proposed a rethinking of the snowmobile avalanche education, as it did not match the snowmobilers needs or dealt sufficiently with complex snowpack management.

Another restraint to avalanche education was stigma issues between skiers and snowmobilers, where snowmobilers were looked upon as “… an outlier, subgroup of the avalanche community until recently” [42] (p. 1139). Being both an avalanche educator and snowmobiler, Predeger pointed out recently that “Recognizing snowmobilers as a specific user group within the official avalanche community in the US, didn’t evolve earlier than a decade ago … So … Leave your Patagonia (ski) gear at home when showing up to a Sledneck event” [44] (p. 26). In other words, not having the audience perspective seems to be a disqualifying factor when the aim is to promote avalanche education that can change peoples’ behavior.

In Norway, Ref. [45] interviewed 10 snowmobilers in Northern Norway and found that their behavior and risk management were not based on any formal avalanche education, but their own local expertise. Since freeriding is illegal in Norway, there are no avalanche courses for this type of snowmobilers and their specific challenges. If they do not attend an avalanche course for skiers, they are on their own, learning by doing.

## 3. Objectives

Our aim was to study the behavior of freeriding snowmobilers in avalanche terrain in Norway, as well as the factors influencing this behavior from their perspective. We raised the following three research questions:

1. What do snowmobilers do and not do in avalanche terrain?
2. How does their behavior relate to managing complex avalanche conditions?
3. Is there a mismatch between avalanche knowledge and riding preferences?

## 4. Method and Material

In this study, a qualitative exploratory approach was chosen since the behavior we wanted to investigate had not been studied in the field in Norway before. Thus, a case-based nonexperimental data collection was beneficial using observational methods in the field [46]. We present the methods, data collection and analysis in the following sections.

### 4.1. Ethnographic Approach

Since we needed data from a specific group and not the general snowmobiler, we chose a non-random participant selection, beneficial in exploratory stages of projects to increase the internal validity [47]. To be able to increase reliability, we sought several data sources to achieve what Burkve [48] and Morse [49] describe as N. Denzin`s source triangulation. We also chose two different focus areas to ensure that the data that were replicated within the focus group were not only valid for one specific area [50]. Our research design took advantage of both insider and outsider perspectives regarding participant communication and data interpretation, since the lead researcher had dealt with avalanche management in the region for 20 years without being a part of the snowmobiling community. Although Strong–Cvetich pointed out in their study that “Qualitative interviews with key members of the mountain snowmobile community might offer more valuable insights to the attitudes and motivations of the hard-to-reach segments of the overall population” [11] (p. 137), we were afraid that structured interviews could feed the hard-to-reach target group with predefined topics with the risk of losing significant audience perspectives.

We chose an ethnographic approach implying unstructured open conversations such as everyday talk. This has proven useful in related research [51,52]. We used phone conversations, email and text messages. In addition to revealing what they did, it was equally important to detect what they did not do or say regarding their avalanche assessment in relation to best practice. To increase reliability, observations and interviews were “member checked” [53] by key participants. This secured their audience perspective and reduced researcher interpretation bias.

Recruitment of key participants was based on initial key participant insider suggestions and snowballing. Random participants were used to verify the key participant data and observations. Random participants were people we would meet by chance in the field during direct observations. As soon as a conversation became relevant, the study was presented, and the participants gave their consent to participate. Semi-structured conversations were used when contacting, e.g., mountain police or resources outside of the illegal snowmobile community. Media contributed with incident reports and public opinions.

Since the target group snowmobiled illegally, practical and ethical issues needed to be solved. A feasibility study [54] revealed that joining the illegal snowmobiling to gain thick descriptions [55,56] would be ethically and practically problematic. Observing from a distance at key locations was considered next. This was ruled out since the researcher had experienced prior this research an increasingly dangerous behavior (show off effect) when meeting snowmobilers as a skier.

Due to the ethical dilemmas, we developed a new method not presented in prior research. We sought to combine insider information using key participants, with field observations of not only of tracks observed, but just as important, where in the terrain there was an absence of tracks. In contrast to recent GPS detection of legal snowmobile tracks in the US [57], this method of observation avoids the risk that participants altered their behavior knowing they were monitored during a research project.

A typical field day would be ski touring, observing where tracks were made and not made, taking pictures and seeking higher terrain to visually observe riding patterns. Tracks would provide different data depending on when the observations were made. Tracks made right after a snowfall provided reliable data regarding riding style preferences and also informed how riders interpreted the avalanche situation in relation to best practice avalanche management. Observations made when there were more accumulated tracks revealed the overarching behavioral patterns, since it became obvious what kind of terrain the riders did not use. Notes would be made on site or immediately after, depending on the weather conditions. Key participants were contacted frequently for interpretation and verification early in the fieldwork.

### 4.2. Data Collection

**Figure 3:**
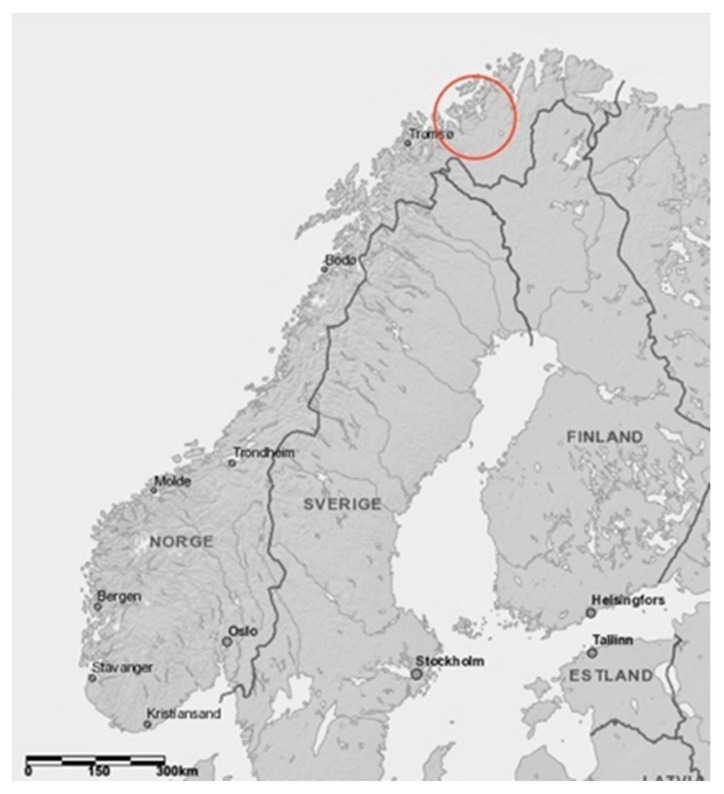
Study area in Northern Norway.

Field data were collected in Northern Norway in the western part of Finnmark County (Figure 3) between January 2018 and March 2019. Supplementary data were collected until August 2019. In the focus areas eight observational field days were performed in January, February, March and April of 2018 and 2019. The key participants (*n* = 7) contributed the same periods as the observations were performed. There were two random participant conversations in one of the focus areas (1b). Conversations (*n* = 6) with external sources outside of the target group, e.g., mountain police, were also conducted during the same periods with one exception. Reports in regional media (*n* = 3) provided pictures of nonfatal incidents in other areas of Finnmark in March and April of 2018.

### 4.3. Data Analysis

The analysis was a reflective cyclical process implying data collection–coding–theoretical sampling–more data collection, resulting in new theory [58,59]. The observational data collection ended in March 2019 when adequacy was achieved [49] and fieldwork only confirmed previously documented codes and categories [60].

Our data evolved from the “bottom-up” through our open-ended research questions. We had an open coding process [61] by identifying codes in our notes, e.g., events and behavior described by key participants. Similar data codes created categories that revealed the themes that are discussed in the paper.

The lead researcher and first coder performed the fieldwork and coded the data first. Next, a second coder coded the data independently. The procedure with a second coder or inter-rater should provide a more consistent interpretation and analysis of the data [50,62]. To ensure the interpretation of the key participant data, the field notes and pictures, it was significant that the primary investigator and the second coder had extensive knowledge of the phenomenon as avalanche professionals and practitioners, providing sufficient situational awareness. We considered an additional external co-coder re-check [63] unnecessary since the second coder was not part of the initial data collection design or coding.

The primary coder and investigator structured the coding in three themes based on eight categories:

- Game changer: 1. new riding style, 2. new challenges, 3. new terrain preferences
- Competence: 4. avalanche knowledge, 5. safety perception
- Consequences: 6. attitudes, 7. monetary consequences 8. accidents

The co-coder identified 21 data blocks, of which 19 matched or fit within the primary investigator’s coding and complemented the categories and themes. We applied Miles and Huberman’s [50] formula to our analysis: # data block agreements (=19)/total # of agreements + disagreements (=21) = 90%. An intercoder reliability score of 90% is regarded as a sufficient agreement score.

## 5. Results

### 5.1. Changed Preferences for Terrain, Snow and Riding Styles

**Figure 4:**
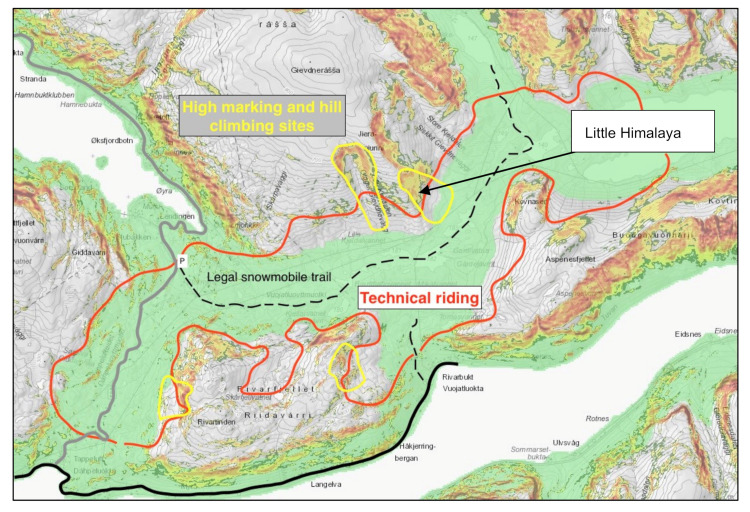
Focus area 1b. Yellow polygons show areas that used to be popular for high-marking and hill climbing. Red polygon shows the area which is now used for technical riding. The polygons are overlaid on a map from www.senorge.no (accessed on 6 January 2022), showing terrain steeper than 30 degrees (yellow to red) and forest/vegetation (green).

Figure 4 illustrates our findings regarding behavior based on our field observations and was commented on and confirmed by key participants within the target group. Most of the illegal technical riding was observed within the red polygon, characterized by sparsely forested terrain and terrain traps. More tracks were observed during periods of deep powder snow conditions than when conditions were compacted by old tracks or strong wind. Tracks were seldom observed in the previously popular high-marking and hill climbing locations (yellow polygons) during our study.

The observed track pattern was explained to us by key participant no. one: “You need more snow in general to do technical riding compared to high-marking that is limited to a loaded lee face. This has changed the preferences-what to do where within the community the past years… (since) you preferably need forest (soft snow) and terrain features to play in”. Our focus areas were confirmed by key participant no. one as the most attractive areas for technical riding in the region. Especially “Øksfjordbotn (1b) is a playground for technical sledders”. The qualities of the area to perform technical riding was also confirmed by key participant no. seven, who told us that sledders travel as far as 300 km from the east of Finnmark to ride 1b. People have also traveled from Finland and Southern Norway to pursue technical riding in the 1b area (key participant no. one). Technical riding has relocated the avalanche-exposed riding within the Alta region due to the deeper snow cover and more frequent snowfalls combined with the terrain features.

Untracked high-marking terrain indicated that high-marking had become less popular (Figure 2) in the area. We observed some high-marking and hill climbing in areas 1a and 1b, but it was limited to smaller terrain features than before. A previously popular five-hundred-meter slope named “Little Himalaya” (Figure 4) had no observed high-marking activity during our field days. This reduction in high-marking activity was confirmed by key participant no. seven. Despite favorable snow conditions with less persistent weak layers, he had not observed high-marking on Little Himalaya during 2019 (nor 2018 when a shallow snow could have explained the reduced activity).

The tracks and new behavior we observed with reduced high-marking and hill climbing were further explained by key participant no. two: “The machines have developed, and it is more fun maneuvering challenging and tricky terrain than high marking straight up”. In addition to revealing the significance of the improved technology that made it more fun when performing technical riding, key participant no. seven told us that there has been a status change among the sledders regarding what is prestigious, and discredited high-marking: “Anyone with enough horsepower can ride the high marking slopes which demands less skills compared to technical riding”. The change in activities from high-marking and hill climbing to technical riding indicated a game changer, as the riders have changed their riding style and terrain preferences significantly.

### 5.2. Concerns for Avalanche Competence and Awareness

Several of our key participants (nos. one, three and seven) expressed concern about the ongoing behavior and how their fellow snowmobilers underestimated risk and exposure. Key participants no. one and seven were disappointed regarding the attitudes and behavior the riders displayed when dealing with persistent weak layer situations (PWL). Key participant no. seven exemplified this concern since he feared that the “Riders would go crazy with an upcoming snowfall” due to a long period of limited riding options in 2018 during a PWL situation with risk of damaging one’s snowmobile.

Key participant no. two acknowledged the new complex terrain trap exposure they had to deal with: “It`s also tricky many times to see the terrain traps in the forest and evaluate the terrain above. There is a lot to consider”. Key participant no. one expressed a competence concern after meeting two random riders who had triggered small avalanches in location 1a, which is known for its hill climbing and high-marking and large PWL avalanches. Having advanced avalanche knowledge as a snowmobiler, he asked how their side hilling (a sled maneuver that cuts across the slope and can dig deep into the snowpack) related to the avalanche situation. They answered that “The avalanches were not dangerous that day, since they were only powder avalanches”. Key participant no. one interpreted their answer as a lack of competence and respect for the dangerous PWL situation and the avalanche forecast. Best practice would have been to avoid avalanche terrain, since small avalanches could propagate or stepdown and cause large fatal avalanches when riding.

None of our key participants expressed that their fellow snowmobilers had sufficient competence, or the awareness needed to assess the avalanche danger, but rather the contrary. A random participant in the field told us about the basic avalanche courses that had been organized in the region. These were initiated within the targeted community, but there was little interest in taking more advanced courses on technical riding and managing complex avalanche situations.

### 5.3. Risk of Accidents, Fines and How Material Damages Influence Behavior

According to key participant no. seven, “There are rumors every season about all the near misses”. Incidents and close calls are never reported officially or shared publicly since the activity is illegal. Filmed avalanche incidents have been posted on social media more or less anonymously. During field work in 2018, key participant no. three described several close calls at location 1a, implying the triggering of avalanches and partial burials. Close calls had an impact on key participant no. two. He mentioned that close calls and accidents when high-marking convinced him to shift to technical riding. Getting older and smarter with increased responsibility in life made him less willing to take the risks he and friends of his took previously when high-marking. Technical riding was presumed safer.

The police in Finnmark and Alta told us that the influence on behavior due to avalanche accidents was disappointingly short lived. There have been several fatal accidents in the region, one of which was west of our focus areas in March 2018, most likely due to technical riding. Pictures provided by the police for this study indicated that the avalanche was remotely triggered by the snowmobiler when riding at the bottom of the forested valley. The accident illustrated the feared combination of technical riding, persistent weak layers and a terrain trap mentioned by key participant no. seven. Our participants did not reveal if there were any attitudinal changes after an accident regarding the use of personal avalanche rescue gear.

What did change behavior was the fear of damaging the machine. We revealed a new risk perception due to shallow snowpack conditions in 2018. Key participant no. one explained the riding as insurance conditions or “Kaskoføre”. The reasoning was that the fear of hitting rocks in the shallow snowpack limited the riding to more densely forested terrain. This forced them into terrain traps where the snow cover was thick enough to maneuver. Different from previous riding, this behavior was motivated by fear of monetary consequences.

Monetary consequences due to the risk of getting fined from police controls did make an impact on when to ride where. After viewing a closed Facebook group that warned members about possible police controls, we observed less tracks than expected after a mid-season weekend with nice weather and recent snowfall.

## 6. Discussion

The study provides an audience perspective of what snowmobilers do in avalanche terrain in Norway using an ethnographic approach to get as close to real life as ethically and practically possible when studying a risk-prone illegal activity. Our study has important contributions to others interested in observing hard-to-reach groups. In this section, we discuss our findings related to behavior, preferences, competence, equipment and education, as well as how our method provided access to data, we most likely would have missed otherwise.

### 6.1. Game Changer

After two seasons of observations, we documented a game changer regarding snowmobiler behavior in Northern Norway, as technical riding was documented for the first time. Our findings are important for education and risk management and suggest that Norwegian snowmobilers face similar challenges as those in Canada. The track patterns we observed were confusing at first but were made understandable thanks to the use of key participants. A new type of technical riding was observed during the first field season, named “insurance riding” by our key participants. A long winter with limited snow the first months made the riders focus on how to ride without damaging their machine due to few options with a shallow snowpack. Those with the right machine and skills could expand their terrain usage to include denser forests, deeper snow and more avalanche terrain traps compared to riders with poorer equipment or skills. This was a significant part of the new terrain usage and increased exposure to avalanche terrain traps with possible fatal consequences.

The missing high-marking tracks were first thought to be due to the previously mentioned very shallow snow cover the first field season. However, high-marking tracks were also missing the second season with a thick snow cover. We argue that the behavioral pattern detected could not be dismissed due to abnormal conditions or as a local phenomenon, since riders from other parts of Norway and neighboring countries visited focus area 1b without leaving high-marking tracks either. The behavioral patterns were further confirmed by Swedish snowmobile researchers [64,65] and a Norwegian target group snowmobiler [66] during their presentations at the Nordic avalanche conference in 2019. We do not claim that no one is high-marking anymore–modern snowmobiles can easily do this—but the observations suggest a behavioral change with significant implications for education, risk management and research.

Warnings of police controlling on a closed Facebook group resulted in less riding in focus area 1b. The snowmobilers obviously wanted to avoid being fined, but law enforcement did not seem to induce any behavioral change, since they just relocated the same behavior. It is likely that similar warning groups are to be found in other parts of Norway with effects on the amount of activity. Based on our findings, the snowmobilers do not alter their preferences due to the consequences of breaking the law.

We argue there are three interlinked reasons for the change in behavior: 1. the technological improvements of the snowmobile (powder snow requirements), 2. desired powder snow skills in complex terrain that replaced the simpler high-marking up and down in specific slopes, 3. the region had areas with frequent snowfalls and preferred terrain. We find similar patterns regarding behavioral change within off-piste or backcountry skiing, where improvements in technology (skis) have made it easier for more and less skilled people to enjoy deep powder conditions and relocate into steeper avalanche terrain more often.

Our findings indicated that social comparison as a strategy for behavioral change was efficient, despite not being an official policy and strategy as in the US [13,15]. In our case, the technical riders in the unofficial illegal community did this without interference by enhancing their self-image as more sophisticated and prestigious compared to high-marking.

Even though the key participants expressed a lower personal risk acceptance after close calls, they could not see that accidents within the target group had significant impact on the overall behavior regarding avalanche safety. This corresponded with the impression passed on to us from the police.

The tracks we observed during PWL periods did not reveal best practice avalanche management. The youngest riders’ social comparison competence buildup seemed to be more about riding skills than avalanche management. The situation in Norway is very different from the US, since there are few public professional role models that the riders could identify themselves with that both portray avalanche safety and riding skills. An avalanche course provider for the target group in our study confirmed the studies in Canada [16,17], where they also found little interest beyond the basic avalanche course. In comparison to skiers, this might be due to the more easily accessible “learning by doing” possibilities, and, just as important, how they look upon what is relevant knowledge buildup, as pointed out by Stewart–Pattersen et al. and Staple [13,16].

### 6.2. Technical Riding, Snow Compaction and PWL Issues

Our study has shown that technical riding has largely replaced high-marking and hill climbing. We have documented that new terrain features have become attractive due to technical riding. This was terrain previously passed on the way to high-marking sites (Figure 4). The new preferences also revealed increased decision-making complexity and uncertainty. This indicates that a misunderstood competence buildup pointed out by Stewart-Patterson and Hanke [20] and Pawliuk [43] seems likely, and not only for those within the local target group that predominantly high-marked before. This seems even more relevant for the snowmobilers that arrived in the area 1b from other parts of the region, Norway and neighboring countries, who were most likely used to more compacted conditions. They might start riding without an update on the avalanche situation, lacking significant local knowledge.

As shown in Figure 4, terrain usage has evolved from being static and more predictable high-marking in a few small areas to become more dynamic, using a much larger area with increased uncertainty and terrain complexity. The previously popular high-marking sites were tracked frequently, with resulting compaction, reduced PWL development and an overall lower likelihood of avalanches. The high-markers could monitor avalanche slopes as ski resorts do [33,36], since the terrain usage was static, the risk was more defined and the riders would typically watch each other. Technical riding has introduced a more dynamic terrain usage. This has implied an increased use of terrain traps, typically around and below the wind-exposed tree line sections connected to avalanche terrain above. This dynamic terrain usage may render riders more vulnerable to a false sense of safety [33]. They may base their second ride on the experience from the first ride performed on a snowpack with no PWL alarm signs due to a fresh snowfall hiding old tracks, thus concluding that it is safe.

Snow compaction research [67] and focus on persistent weak layer management [68] have primarily been for skiers [33]. Since technical riding implies a more spaced-out track pattern that will reduce the stabilizing effect of the snowmobile maneuvers [35], the ongoing work in Canada by Stewart-Patterson, Exner and Hanke [35] on the effects of snowmobile maneuvers on the snowpack will hopefully provide management recommendations we can adjust to Norwegian conditions. The rider’s awareness of increased uncertainty since the slopes were not tracked frequently might explain why we did not see high-marking tracks in the most popular sites. They might have known that decision expertise developed in tracked areas is not transferable to untracked snowpacks. More research is needed to grasp why they do as they do in this regard. Our findings support prior research on snowmobilers’ competence regarding PWLs and complex snowpack situations [22] by revealing a low perception of PWLs and the importance of having a relocation strategy when in elevated danger. We also see that education needs to put more emphasis on misunderstood competence, false sense of safety and terrain trap management [13].

### 6.3. Mismatch between Preferred Snowmobiling, Competence and Education

The mismatch between the preferred snowmobile riding and education in Canada developed since new machines made it easier for less knowledgeable and less experienced riders to access more terrain and complex situations. How is this in Norway? After answering the first research question we learned that our target group has the new machines as in Canada. We learned from observations and key participant statements that they prefer technical riding, access more terrain and end up in more complex situations than before. Track observations and insider concern voiced by key participants suggest that lack of respect for PWL conditions results in annual close calls and fatal accidents.

The mismatch in education and preferred riding is obvious in Norway, as there is no avalanche education for our targeted snowmobilers since their behavior is illegal. Thus, our target group has to organize courses themselves, attend courses for skiers without snowmobile-specific issues, learn from YouTube or just continue learning by doing. As in Canada, the target group had little interest in advanced courses.

What we do not know is if the riders have had an uncritical transformation of high-marking management skills to technical riding, and this could result in the underestimated risk recognition emphasized in previous research [22]. One of our key participants did tell us that he shifted from high-marking to technical riding because he presumed technical riding to be safer. Key participants also expressed concern for their friend’s behavior after a period of compacted snow with limited riding options. To obtain a better impression of the riders’ avalanche knowledge, participant observation may be the preferred option to disentangle the riders often high riding skills and practical experience from their avalanche assessment skills.

This study found the same mismatch between education and behavior previously described in Canada. To remedy the situation, one of our key participants suggested that future education efforts should target the younger riders, who have the newest machines and highest riding skills, but the least experience.

### 6.4. Ethical and Practical Constraints and the Need for a New Tool

There is frustration among ethnographers regarding rigid ethical standards making observations in public spaces practically impossible [69]. This challenge became even more evident in our study since the behavior we wanted to observe was illegal. By changing the observational strategy from people to the tracks they made and did not make, we found the tool that did the job. Ethical safety concerns were avoided for both the researcher and participants in our study, as the researcher did not have to join the avalanche-exposed activity or risk influencing the participant behavior. Privacy and anonymity were preserved, since the old tracks did not reveal anyone, and they told the truth. Since we did not meet the key participants in the field, we avoided rumors and distrust within the community that could have influenced behavior. In addition, we wanted the participants to talk about the behavior of “others” to detect broader behavioral patterns. Despite the challenges, we argue that our ethnographic approach and method revealed a new option for research on hard-to-reach groups and studying behavior in a risk-exposed environment.

### 6.5. Limitations

Caution should be used when generalizing our findings beyond our case area. Hopefully, our research and field method can fuel similar research in other parts of Norway, since answers from the general snowmobiler that avoids avalanche terrain are not the data of interest. The method we used did not capture the riders’ avalanche knowledge level, as presented in surveys [10,22,42]. Qualitative interviews with key members, as suggested by Strong–Cvetich [11], would most likely have provided more in depth insights regarding competence level, decision making and attitudes. Participant observation would have given a better impression of the riders’ avalanche assessment and decision-making competence. The GPS tracking applied by Hendrikx and Johnson [57] could have provided a more accurate interpretation of how terrain usage correlated with avalanche problems. Since the riding was illegal, we had to disregard this option since we feared that it would influence their behavior knowing that they were monitored. Awareness of biases on how the researchers could influence the fieldwork and analyzation procedure was essential [56]. The lead researcher’s background as an avalanche course provider in the region could influence key participants to comply with the researcher and answer what he wanted to hear. Since we could combine the tracks with random participants not acquainted with the researcher, we hopefully cross checked the essential information and reduced this potential bias.

## 7. Conclusions

We studied the behavior of freeriding snowmobilers in avalanche terrain in Norway and the factors influencing this behavior from their perspective. Prior to this study, a common belief was that high-marking caused many of the snowmobile avalanche accidents. Our field observations, confirmed by key participants over two seasons, revealed that technical riding in a more complex environment has become more popular due to the technical evolution of snowmobiles and social comparison dynamics within the targeted group.

The transition to technical riding implies more challenging risk management, with increased complexity in terms of snow and terrain traps. The mobility and attitude of the riders raises concerns about limited self-competence and a false sense of avalanche safety, in particular during snow conditions with persistent weak layers. The situation in Norway resembles Canada, with a possible mismatch between education and actual behavior. The lack of insider snowmobile avalanche safety mentors in Norway is a major challenge if social comparison strategies are to be considered. It seems clear that the effect of existing policy and law enforcement is limited in terms of changing snowmobilers’ riding preferences or increasing their avalanche knowledge level.

This study of a predominantly illegal activity also sheds light on how to explore and observe a hard-to-reach target group. The methods developed should be of interest for a wider audience in other research disciplines.

If we had only observed tracks on flat or gently sloping forested terrain, we would not have any snowmobile avalanche accidents in Norway. However, this was not the case. We fear that the observed behavioral changes are a recipe for more accidents in the future due to the mismatch between education, competence and behavior as more people with limited experience, skills and knowledge with avalanche risk management obtain easier access to more complex situations.

## Data Availability

The qualitative data in this project are of a nature where it is impossible to fully anonymize them. We do therefore not provide the data openly available.
